# Overall gestational weight gain mediates the relationship between maternal and child obesity

**DOI:** 10.1186/s12889-019-7349-1

**Published:** 2019-08-07

**Authors:** Michele J. Josey, Lauren E. McCullough, Cathrine Hoyo, ClarLynda Williams-DeVane

**Affiliations:** 1 Arnold School of Public Health, Department of Epidemiology & Biostatistics, University of South Carolina, 915 Greene Street, Columbia, SC USA; 2 Biomedical and Biological Sciences Department, Bioinformatics Genomics Computational Chemistry Core, Biomedical/Biotechnology Research Institute, North Carolina Central University, 1801 Fayetteville Street, Durham, NC USA; 3 Rollins School of Public Health, Department of Epidemiology, Emory University, 1518 Clifton Rd, NE, Atlanta, GA USA; 4 Department of Biological Sciences, Integrated Health Sciences Facility Core, Center for Human Health and The Environment, Epidemiology and Environment Epigenomics Laboratory, North Carolina State University, Raleigh, USA; 5 Department of Mathematics and Computer Science, Fisk University, 1000 17th Ave, Nashville, TN USA

**Keywords:** Obesity, Gestational weight gain, Child obesity, Mediation, Causal inference

## Abstract

**Background:**

Approximately 17% of children in the U.S. are obese. Children that are overweight or obese are also more likely to be obese as adults and suffer from various chronic diseases and premature death. Maternal obesity can affect the weight status of her offspring through intrauterine mechanisms like excessive gestational weight gain (GWG). Current literature shows a positive association between maternal weight status and GWG on child obesity, yet the direct and indirect effects have not been decomposed or quantified. The purpose of this study was to estimate the effect of maternal obesity on child obesity, mediated by GWG, which is a modifiable risk factor.

**Methods:**

The study participants were a birth cohort of offspring from women who received prenatal care in the Duke/Durham Regional health care system in Durham, NC between 2005 and 2009. Anthropomorphic data was collected via electronic medical records (EMRs) during each voluntary visit to a health care facility. The exposure of interest was maternal obesity, measured by pre-pregnancy body mass index, the mediator was GWG, dichotomized into excessive and not excessive based on maternal prenatal BMI, and the outcome was child obesity at age 4, measured as BMI z-scores from the last recorded height and weight. A counterfactual theory-based product method analysis estimated the mediated effects of GWG, adjusted for maternal race, socioeconomic status, and smoking status.

**Results:**

Of the 766 children, 25% were overweight or obese, and among all mothers, 25 and 31% were overweight and obese, respectively. Maternal BMI was associated with an overall increase of 0.04 in offspring z-score. The proportion of the effect of maternal obesity on child age 4 obesity mediated by GWG was 8.1%.

**Conclusion:**

GWG, in part, mediated the relationship between maternal BMI and childhood adiposity. Even when the mediator is fixed, children are at an increased risk of a higher BMI if the mother is obese. These findings highlight an important public health education opportunity to stress the impact of a pre-pregnancy weight and excessive GWG on the risk of child obesity for all mothers.

**Electronic supplementary material:**

The online version of this article (10.1186/s12889-019-7349-1) contains supplementary material, which is available to authorized users.

## Background

Approximately 17% of children aged 2–19 years in the United States are obese [1], and the rate of growth of the number of obese children has been considered an epidemic [2]. In addition to the social stigma [3], childhood obesity causes insulin resistance, hypertension, fatty liver disease, and other cardiovascular related diseases in adulthood [4]. Children that are overweight or obese are also more likely to become obese adults [5], which introduces additional risks for various chronic diseases like cancer, heart disease, type 2 diabetes, and premature death [6, 7]. Without increased attention to this epidemic, health care expenditures attributable to obesity are projected to increase by at least $48 billion per year by 2030, with obesity accounting for a loss of approximately 2 million productive person-years in working US adults [8]. Therefore, it is imperative to identify modifiable risk factors to reduce childhood obesity and its future consequences.

Obesity has a complex etiology, which extends beyond energy imbalances. Excess body weight has been associated with genetic variation and epigenetic response to environmental cues during intrauterine and early development, including, gestational diabetes mellitus (GDM), high maternal adiposity, birth weight, and poor diet and exercise behaviors [9–11]. For example, obese and morbidly obese mothers are at increased risk of delivering a large-for-gestational age infant [10, 12]. Elevated birth weight negatively affects the offspring weight status trajectory into childhood and adolescence [13], where children with higher birth weights have a higher likelihood of becoming overweight or obese [14, 15]. Also, obese and morbidly obese women, have higher odds of developing GDM [16]. During gestation, insulin resistance in overweight and obese women is further exaggerated which results in maternal hyperinsulinemia, hyperglycemia and GDM [17]. GDM is associated with an increased risk of overweight or obesity in offspring [18]. Even without preexisting diabetes, obese women are still more likely to develop GDM [18].

In addition to a higher birth weight and GDM, pregnant overweight and obese women tend to exceed the Institute of Medicine (IOM) gestational weight gain (GWG) recommendations compared to normal-weight women [19–21]. Evidence from cohort studies show that exceeding the IOM weight gain recommendation is associated with higher BMI percentiles and increased risk of overweight or obesity in offspring [22–25]. In addition, these studies suggest that intrauterine exposure to maternal obesity may induce child obesity risk via dysregulated-appetite, metabolism, and activity levels [24]. For example, a high pre-pregnancy BMI along with excessive GWG increases the risk of having a baby that is large for gestational age [19, 26], and overweight or obese in childhood [25].

### Mediation

**Fig. 1:**
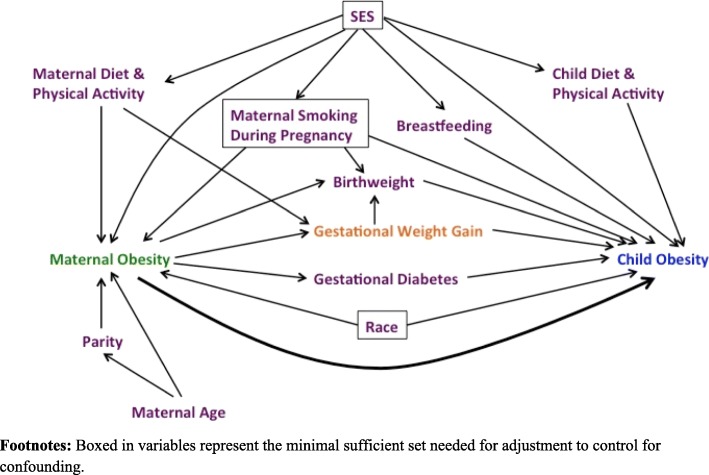
Directed acyclic graph of hypothesized causal relationship between maternal obesity and child age 4 obesity. Boxed in variables represent the minimal sufficient set needed for adjustment to control for confounding

When studying the relationship between maternal and child obesity, previous literature has considered GWG to be a confounder to be adjusted for [27] or interaction term [19, 21, 25, 26, 28]. These studies showed evidence of a synergistic effect of adverse maternal and child outcomes when the mother has both a high BMI and gains excessive weight during pregnancy. However, there is little research on quantifying the direct and indirect mechanism of maternal obesity on child obesity, mediated through GWG. Current literature consistently shows a relationship between maternal pre-pregnancy obesity, GWG, and later child obesity [21, 29], as depicted in Fig. 1(SAS Code included in Supplemental Material). In this figure, the thick arrow illustrates the direct effect of maternal pre-pregnancy obesity on child obesity, and the arrows from maternal pre-pregnancy obesity to GWG to child obesity illustrate the indirect, mediated effect. This mediated path is of interest because it has not been previously quantified and GWG is a modifiable risk factor, which presents an opportunity for public health intervention.

The purpose of this study was to estimate the casual effect of maternal obesity on child obesity, mediated through gestational weight gain. We hypothesize that the indirect effect of maternal obesity on child obesity through GWG is positive.

## Methods

### Study population

**Fig. 2:**
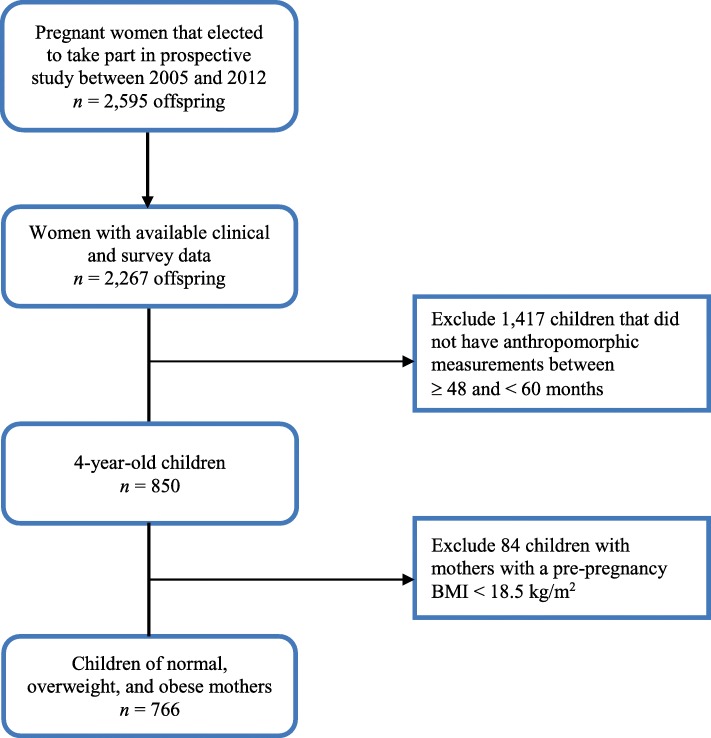
Flow chart of cohort progression from study start to sample selection. Footnotes Boxed in variables represent the minimal sufficient set needed for adjustment to control for confounding

The study participants were the offspring of 2595 women who received prenatal care in the Duke or Durham Regional health care system in Durham, NC between 2005 and 2009. Only women 18 years of age and older with the ability to speak English were eligible for this study. Women were excluded if they did not intend to have custody of the child, had HIV, or planned to receive obstetric care outside of Duke or Durham Regional Hospitals, or planned to move out of the area. Only children of enrollees with available clinical and survey data were considered for this study (*n* = 2,267). The offspring of the women formed an ongoing, longitudinal birth cohort. Figure 2 shows the progression of the full cohort to the sample selected for this study. Anthropomorphic data from the cohort was collected via electronic medical records (EMRs) during each voluntary visit to a health care facility. Because the children belong to an open cohort that uses EMRs, the age of the last recorded measurement varies. Some children in the cohort had not reached age 4, and others did not visit a study health care facility between the ages of 48 and 59 months (*n* = 1,417). These children were very similar to those included in the study (*p* > 0.05). Less than 10% of women (*n* = 86) that were considered underweight were also excluded from the analysis, as their offspring experience a separate set of adverse outcomes [30, 31].

### Exposure

Pre-pregnancy obesity, measured as continuous pre-pregnancy body mass index, was calculated using self-reported height and weight in kilograms per square meter of height (kg/m^2^). Weight was measured three months before gestation and height measured at the first office visit. In *n* = 269 women who visited Duke University Medical Center six month prior to pregnancy, the correlation between self-reported and clinic measured weight was high, (rho = 0.99, *p* <  0.0001).

### Mediator

The mediator of interest was GWG. It was computed during each clinic and study visit as the difference between the self-reported weight at last menstrual period and last weight measured up to 7 days before delivery, including the delivery visit. The clinic used the Tanita scale and stadiometer to measure weight and height—these are accurate within 0.1 pounds (lbs) and 1/8 of 1 in., respectively. Categories for GWG were inadequate, adequate, and excessive. Adequate GWG was based on the Institute of Medicine (IOM) guidelines by BMI status of 28–40 lbs. for underweight women, 25–35 lbs. for ideal weight women, 15–25 lbs. for overweight women, and 11–20 lbs. for obese women. Studies have shown that the amount of weight gained during pregnancy is linearly associated with child BMI z-score, (i.e. higher GWG means higher child BMI) [22, 32]. Therefore, the final GWG variable was dichotomized into excessive and not excessive weight gain.

Valeri and VanderWeele (2013) introduced a mediation analysis method using the counterfactual approach to account for confounders in order to estimate the controlled direct, natural direct, and indirect effects [33]. In context, the natural indirect effect (NIE) is the average difference in child BMI had all the women been of normal weight and then gained excessive gestational weight versus all women being normal weight and gained non-excessive gestational weight. The natural direct effect (NDE) is the average difference in child BMI had all the women been obese and gained non-excessive weight versus all women of normal weight and gained non-excessive gestational weight. Non-excessive gestational weight gain is the category of GWG that one would expect a normal-weight mother to gain. The controlled direct effect (CDE) is similar to the NDE when mothers gain a specific amount of weight during pregnancy. This method is also able to capture the potential interaction between the exposure and mediator, which describes the relationship between maternal obesity and GWG. Assumptions for a causal mediation analysis are: no unmeasured confounding between the exposure and outcome, exposure and mediator, mediator and outcome, and none of the mediator-outcome confounders are induced (or caused) by the exposure. The assumptions were satisfactory for our research aim and data.

### Outcome

The outcome of interest is child obesity at age 4. Body size was measured using the continuous BMI age- and sex-specific, standardized z-scores. The z-scores were calculated by first computing the traditional BMI of weight (kg)/height (m)^2^, then transformed according to the 2000 CDC growth curve for children aged 2 to 19. The age, height, and weight of child were recorded during each visit, resulting in multiple follow-up measurements per year. Only children that reached age 4 were eligible for inclusion, and the last recorded height-weight pair was selected to calculate the BMI when the child was between 48 and 59 months.

### Confounders

Maternal race, socioeconomic status (SES), gestational diabetes, and smoking status was measured via self-report at baseline before delivery. These variables made up the minimally sufficient set needed for adjustment obtained from a causal directed acylic graph (Fig. 1), a planning tool used to reduce bias in epidemiologic studies [34]. Highest educational attainment served as a proxy for SES. Smoking status was dichotomized into no smoking during pregnancy and smoking for any period during pregnancy. In addition, a cohort variable was included to account for any differences in the enrollment periods, if present. Most children were enrolled between 2006 and 2008 or between 2009 and 2011. Additional parturition data, including maternal age, delivery mode, gestational age, infant sex and birth weight, were extracted via EMRs, and were included in a separate sensitivity analysis. Pregnancy complications, like gestational diabetes, were assessed during prenatal visits and EMRs.

### Statistical analysis

The mediation analysis utilized the modified product method approach that allows for additional covariates and interaction described by Valeri and VanderWeele [33] in order to estimate the direct effect of the exposure on the outcome, as well as the effect the exposure on the outcome mediated through GWG. The model included the confounders from the minimal sufficient set. Logistic regression was used to estimate the effect of the exposure on the mediator, and linear regression to estimate the effect of the exposure and mediator on the outcome. Pre-pregnancy BMI was considered exposed at BMI of 30 and unexposed at BMI of 22. The parameter estimates from the two models were used to estimate the controlled direct effect (CDE), natural direct effect (NDE), and the natural indirect effect (NIE).

A sensitivity analysis was conducted to assess the validity of the chosen model using a fully adjusted model with all confounders including parturition and maternal clinical data. Standard errors were calculated using bootstrapping with 1000 samples within the mediation macro to estimate the direct and indirect effects of the exposure on the outcome (see Supplement). Child height and weight data were cleaned using the MonoInc package in the R programming language prior to calculating the BMI [35]. The MonoInc package identified data that departed from the monotonic pattern, then imputed using two methods, Last & Next (average of previous and next value) and fractional linear regression. The final value was a weighted average (0.3 and 0.7) of the imputed values. Multiple imputation using FCS discriminant method for arbitrary missing patterns was employed to produce more reliable estimates by imputing missing covariate data [36]. Bivariate comparisons of children by weight status were made using a t-test or Chi-square test for continuous and categorical variables, respectively. These remaining analyses were carried out in SAS 9.4.

## Results

There were a total of 766 (34% of cohort) mother-child pairs in the analyzed dataset. The children had a mean (sd) follow-up time of 63.2 (15.9) months. Approximately 25% of all the four-year-old children were overweight or obese, and among all mothers 25 and 31% were overweight and obese, respectively. Compared to normal and underweight children, overweight/obese children were more likely to have mothers that were obese (27% vs. 44%), who gained an excessive amount of weight during pregnancy (53% vs. 65%), and had lower educational attainment (*p* = 0.013); see Table 1.

**Table 1:** Child and maternal characteristics of 4-year-old offspring by child obesity status, *n* = 766

Variable	Normal or Underweight	Overweight or Obese	*p*-value^b^
n (%)	581 (76)	185 (24)	
Child Characteristics			
Follow-up time (months)^a^	63.5 (12.3)	62.2 (12.8)	0.295
BMI z-score^a^	−0.03 (0.70)	1.54 (0.40)	< 0.001
Males, n (%)	318 (53)	113 (54)	0.821
Birth weight (g)^a^	3165 (657)	3240 (615)	0.121
Maternal Characteristics			
Age at delivery (yrs)^a^	28.7 (6.2)	28.9 (6.3)	0.970
Education			0.013
Less than high school	109 (20)	50 (28)	
High school Diploma/GED	112 (21)	44 (24)	
Some College	122 (23)	42 (23)	
College Degree	195 (36)	45 (25)	
Race			0.008
Black	257 (47)	87 (47)	
White	185 (34)	50 (27)	
Hispanic	78 (14)	44 (24)	
Other	26 (5)	4 (2)	
Body Mass Index (BMI)^a^	27.2 (7.21)	30.9 (8.4)	< 0.001
BMI Status			< 0.001
Normal (18.5–24.9 kg/m^2^)	266 (49)	56 (30)	
Overweight (25–29.9 kg/m^2^)	134 (25)	47 (25)	
Obese (≥ 30 kg/m^2^)	146 (27)	82 (44)	
Gestational Weight Gain			0.018
Less than Adequate	107 (20)	35 (19)	
Adequate	132 (24)	29 (16)	
Excessive	291 (53)	117 (63)	
Smoked during Pregnancy	117 (22)	47 (26)	0.205
Gestational Diabetes	39 (8)	10 (6)	0.527
Cesarean Section	196 (36)	80 (43)	0.114
Gestational Age (weeks)^a^	270 (17)	270 (13)	0.722
Parity			0.357
0	129 (36)	58 (32)	
1	179 (34)	61 (33)	
2	100 (19)	35 (19)	
≥ 3	62 (11)	30 (16)	
Ever Breastfed	440 (82)	150 (82)	0.999

Footnotes: ^a^ Continuous variable; mean and standard deviation is presented

^b^ *p*-values are based on t-tests for continuous and Chi-square tests for categorical variables

Child BMI categories from BMI z-scores: Underweight (z < 5th percentile), Normal (5th ≤ z < 85th percentile), Overweight (85 ≤ z < 95th percentile), Obese (z ≥ 95th percentile)

Overall, elevated maternal pre-pregnancy BMI increased the risk of offspring obesity [β = 0.04, 95% CI (0.02, 0.06)]. The CDE of obese maternal pre-pregnancy BMI increased offspring BMI z-score by 0.32 [95% confidence interval (CI): 0.20, 0.44] compared to a normal weight BMI. The NDE of maternal pre-pregnancy BMI increased offspring BMI z-score by 0.23 [95% CI (0.16, 0.31)]. The NIE mediated through GWG increased offspring BMI z-score by 0.02 [95% CI (0.003, 0.04)]. The proportion of the effect of maternal obesity on child age 4 obesity mediated by excessive gestational weight was 8.1%.

The results presented in Table 2 only used the minimal set of variables needed to control for confounding. In a sensitivity analysis, a fully adjusted model with additional parturition and maternal clinical data was included (see Additional file 1: Table S1). Even with the additional covariates, natural direct and indirect effects were consistent, though slightly attenuated with overlapping confidence intervals.

**Table 2:** Decomposition of effects of maternal obesity on child obesity, with gestational weight gain as the mediator

Decomposition of effects	Estimate^a^	95% Confidence Interval
CDE	0.322	(0.207, 0.439)
NDE	0.235	(0.157, 0.313)
NIE	0.020	(0.003, 0.042)
Total effect	0.246	(0.165, 0.329)
Proportion mediated (%)	8.13%	(1.82, 12.8%)

*CDE* Controlled direct effect, *NDE* Natural direct effect, *NIE* natural indirect effect

Footnotes: ^a^ The estimated average difference in child BMI z-score adjusted for maternal race, education (SES), gestational diabetes, and smoking status during pregnancy

Total effect = NDE + NIE; Proportion Mediated = NIE/Total Effect

## Discussion

This study examined the effect of maternal BMI on the BMI of their offspring at age 4, mediated by gestational weight gain. Overall, women with a higher BMI experienced an increased risk of having a child with a higher BMI at four years old, even with appropriate GWG. This is reflected in the CDE; it represents the average difference in offspring BMI z-score at age 4 between a normal weight mother (BMI = 22) and an obese mother (BMI = 30), when she does not gain excessive weight. Even when the mediator is fixed, children are at an increased risk of a higher BMI if the mother is obese. Child BMI z-scores are sensitive to small changes, where tiny increases can push an overweight child into obesity.

The results from this study are consistent with previous literature. There is a documented positive relationship between normal weight women that exceed the GWG recommendations and child obesity for similar age groups [19, 37]. Our study was able to quantify the strengths of paths through which elevated child BMI occurs. When considering the impact of GWG, excessive weight gain in both early and late periods of pregnancy were associated with a higher risk of age three childhood obesity measured through BMI z-scores [38]. While our study does not contain information about the timing of GWG, overall excessive weight gain was also found to be associated with an increase risk in the aforementioned study.

These findings highlight an important public health education opportunity to stress the impact of excessive GWG on the risk of child obesity for all mothers, independent of their pre-pregnancy weight status. Currently many maternal health interventions target overweight or obese women for GWG messaging related to healthy pregnancies [39, 40]. Overweight and obese women are advised to lose weight before pregnancy in order to reduce the adverse effects for herself and the child [41]. For example, Kral et al. found that children of women conceived after weight loss surgery were less likely to be overweight or obese compared to women that conceived before weight loss surgery [42]. In essence, moving to a healthier weight before pregnancy helped prevent obesity for their children. Yet, 51% pregnancies are unplanned in the United States [43], which makes losing weight prior to pregnancy impracticable for a large portion of the population. However, gaining the appropriate amount of weight during pregnancy is a modifiable risk factor that can benefit normal, overweight, and obese women. Albeit small, gaining a healthy gestational weight has the potential to eliminate 8.1% of the effect of maternal pre-pregnancy obesity. This small impact is magnified when considering that there were approximately 3.5 million births in 2014, where 26 and 25% were born to overweight and obese women, respectively.

### Limitations

There are limitations that should be considered. Variables associated with obesity, such as diet and physical activity, were not available in this dataset. The DAG constructed for this study does not indicate that maternal or child diet and exercise are confounders that need to be adjusted for in the models. In fact, several studies have found that physical activity and diet are strongly associated with age and SES, both of which were included in the model [44–46]. In addition, our sample size was relatively small compared to other studies. This was partly due to the nature of how the data was collected (i.e. EMRs). However, the follow-up time did not differ by maternal or child weight status at age 4, and the prevalence of child obesity at age 4 and adult obesity are consistent with the national averages [1]. In addition, a study in the Ohio WIC population with a large sample size found a significant association between maternal and child obesity, but did not find a significant effect of GWG on child age 4 obesity status [27]. Despite a smaller sample size, this study was able to find a significant association between maternal BMI, GWG, and child obesity. Also, a common concern is that self-reported pre-pregnancy weight from which BMI is computed may be under-reported due to recall or social desirability bias. We assessed this possibility and showed, in 260 pregnant women, that self-reported weight and height were highly (99%) correlated with clinic measures done within 3 months of self-reports, and 92% in those reporting within 6 months (reference Bernard Fuemmeler, 2016).

### Strengths

The strengths of this study include a racially-diverse population, a longitudinal birth cohort with almost complete maternal data that establishes a temporal relationship between the outcome and exposure. This study also utilized innovative methods to quantify a causal association between maternal and child obesity. Gestational weight gain mediated 8.1% of the effect of maternal BMI on child BMI. To our knowledge, the mediated relationship between maternal and child BMI has not been previously quantified. Even with a significant direct effect of the exposure on the outcome, there was still a significant positive effect mediated through GWG.

## Conclusions

In conclusion, this study sought to quantify the causal effect of maternal obesity on childhood obesity, mediated through GWG. Both maternal BMI and GWG increased the risk of later childhood overweight or obesity at age 4, and GWG only slightly mediated the relationship between maternal and child obesity. Thus, public health messaging should continue to prioritize recommendations about maintaining a healthy body weight, regardless of pregnancy status.

## Additional file

Additional file 1: **Table S1.** Decomposition of effect. (DOCX 58 kb)

## Data Availability

The datasets generated and/or analyzed during the current study are not publicly available due to concerns regarding compromising individual privacy.

## Abbreviations

BMI: Body mass index
GWG: Gestational weight gain
CDE: Controlled direct effects
NDE: Natural direct effects
NIE: Natural indirect effects
DAG: Directed acyclic graph
