# Supplementary material for: Overall gestational weight gain mediates the relationship between maternal and child obesity
Source: BMC Public Health. 2019 Aug 7;19:1062. doi: 10.1186/s12889-019-7349-1 (PMC6686549; doi:10.1186/s12889-019-7349-1)
Supplement: Supplementary file 1 — Table S1. Decomposition of effect. (DOCX 58 kb) [file 12889_2019_7349_MOESM1_ESM.docx]

Additional file 1

**SAS Macro**

%mediation(data, yvar = bmi_z ,avar= mat_bmi ,mvar = gest_wt_gain ,cvar = educ1 educ2 educ3 smoking race1 race2 race3 cohort, a0 = 22 ,a1 = 30 ,m = 0 ,nc = 8 , yreg = linear ,mreg= logistic, interaction= false, output= full , boot=true)

run;

**Table S1.** Decomposition of effects

| **Decomposition of effects** | **Estimate^a^** | **95% Confidence Interval** |
| --- | --- | --- |
| CDE | 0.200 | (0.119, 0.278) |
| NDE | 0.200 | (0.119, 0.278) |
| NIE | 0.010 | (0.0002, 0.0245) |
| Total effect | 0.210 | (0.128, 0.290) |
| **Proportion mediated (%)**^b^ | **4.76%** |  |
| **Footnotes:** CDE: controlled direct effect, NDE: natural direct effect, NIE: natural indirect effect  Total effect = NDE + NIE  ^a^ The estimated average difference in child BMI z-score.  ^b^ Proportion Mediated = NIE/Total Effect | | |
